# Discovery and Potential Utility of a Novel Non-Invasive Ocular Delivery Platform

**DOI:** 10.3390/pharmaceutics15092344

**Published:** 2023-09-19

**Authors:** Weizhen (Jenny) Wang, Nonna Snider

**Affiliations:** Department of R&D, JeniVision Inc., Irvine, CA 92617, USA

**Keywords:** AMD, cornea, docosahexaenoic acid (DHA), drug delivery, non-invasive ocular delivery platform (NIODP), ocular disease, oily eye drops, omega 3, retina, retinal delivery, retinal diseases

## Abstract

To this day, the use of oily eye drops and non-invasive retinal delivery remain a major challenge. Oily eye drops usually cause ocular irritation and interfere with the normal functioning of the eye, while ocular injections for retinal drug delivery cause significant adverse effects and a high burden on the healthcare system. Here, the authors report a novel topical non-invasive ocular delivery platform (NIODP) through the periorbital skin for high-efficiency anterior and posterior ocular delivery in a non-human primate model (NHP). A single dose of about 7 mg JV-MD2 (omega 3 DHA) was delivered via the NIODP and reached the retina at a Cmax of 111 µg/g and the cornea at a Cmax of 66 µg/g. The NIODP also delivered JV-DE1, an anti-inflammatory agent in development for dry eye diseases, as efficiently as eye drops did to the anterior segments of the NHP. The topical NIODP seems to transport drug candidates through the corneal pathway to the anterior and via the conjunctiva/sclera pathway to the posterior segments of the eye. The novel NIODP method has the potential to reshape the landscape of ocular drug delivery. This is especially the case for oily eye drops and retinal delivery, where the success of the treatment lies in the ocular tolerability and bioavailability of drugs in the target tissue.

## 1. Introduction

Tolerability and suitability issues can limit the use of various materials or delivery routes for ocular drug administration, particularly for oily eye drops and retinal drugs. While eye drops are very often used to treat ocular surface diseases, intravitreal injections or implants are mainly used to treat posterior ocular diseases. Since Vitravene^®^ (fomivirsen sodium) became the first FDA-approved intravitreally injected therapeutic agent in 1998, the ophthalmic pharmaceutical industry has been in urgent need of a breakthrough in ocular drug delivery. 

Topical eye drop administration is well known for its capacity to provide necessary and efficient pharmaceutically effective doses to most anterior ocular tissues, usually with higher local drug levels than oral administration and minimal systemic exposure and side effects [1,2]. While most active pharmaceutical ingredients (APIs) are lipophilic, commercialized eye drops are often used in a more ocularly tolerable aqueous formulation that may be subjected to fast elimination by tears compared to an oily formulation. Eye drops containing castor oil as a vehicle have been found to cause corneal toxicity. This is not observed with the use of Aleurites, camelina, maize, and olive oils, suggesting that oil formulations may still be useful as ophthalmic solutions [2]. Although oily eye drop formulations may increase the solubility and pharmacologic effects of the drug, they often cause ocular discomfort, such as vision blurring and foreign body sensations, due to the high viscosity and refractive index of oily contents [2,3]. This is the main limiting factor for their use as eye drop formulations. 

Posterior ocular drug delivery is a major challenge due to the complex anatomy and the dynamic physiological barrier of the eye, which significantly affect the availability of treatments. The physicochemical properties of drugs, formulations, delivery systems, and routes of administration are major factors affecting intraocular bioavailability; they have been explored to enable effective ocular drug delivery [4]. Oral administration and eye drops are the two currently available non-invasive routes of delivery used to treat ocular diseases. Due to the blood–retina barrier, the retinal bioavailability of drugs via oral administration is extremely low and comes with very high unnecessary systemic exposure. In individual baboon neonates, it was demonstrated that the absorption of DHA to the retina from blood circulation reached a plateau when DHA in plasma or red blood cells (RBCs) was approximately 6% of the weight of total fatty acids in the diet [5], meaning that DHA in dietary supplements beyond a certain limit may not be absorbed into the retina. Additionally, the delivery efficiency of DHA from the diet to RBCs in baboon neonates was mostly between 0.013% and 0.04% of the DHA dose, while the correlated diet-to-retina DHA delivery efficiency was calculated to be in the range of 0.0003% to 0.001% [6]. Topical eye drops, a more direct route with lower systemic exposure, have also been shown to be very inefficient for posterior segment delivery. Only <3% of the dosed drug amount in eye drop formulations reached the aqueous humor, and 0.001% or less reached the retina [7,8]. Thus, for the retinal delivery of DHA, the oral route requires megadose systemic exposure, and oily eye drops cause intolerable adverse ocular effects, meaning that both methods are highly inefficient.

While orally administered drugs reach the retina through the blood circulation, the use of topical eye drops is believed to channel drugs to the posterior ocular segments via two possible routes: (1) the corneal route, where the drug penetrates the corneal surface and continues to diffuse through anterior ocular tissues (aqueous humor, lens/iris/ciliary body) to the vitreous humor and may then reach the retina; and (2) the conjunctiva/scleral route, where a drug diffuses from the conjunctiva of the ocular surface through the scleral water channels/pores to reach the retina [7,9]. The concentration gradient of dissolved drugs is the driving force for molecules to permeate through the lipophilic membrane barriers of the eye (i.e., the conjunctiva and/or cornea) along the corneal and scleral pathways [7,10]. For a drug with favorable physicochemical properties (e.g., molecular weight, radius, charge, and lipophilicity), the scleral pathway provides a bypass around the anterior segment barriers (the lens, iris, and ciliary body), allowing drug permeation to the back of the eye [11,12]. 

Although many rabbit and rodent studies claim to have achieved successful retinal delivery by applying high doses of small molecules or proteins in eyedrop formulations, successful translation to larger species or clinical success has not yet been achieved, and topical eye drops remain highly inefficient for posterior segment delivery [13,14,15]. In these rabbit and rodent studies, the amount of therapeutic agents reaching the retina was mostly in the range of a 0.01 to 0.1 µg/g concentration after a typical eye drop regimen [13,16,17,18,19]. In such circumstances, retinal delivery due to systemic exposure cannot be ruled out because of the small body volume of these animal models. Therefore, rabbit and rodent models may not be appropriate for studies of posterior segment drug delivery [13].

The current standard of care for retinal drug delivery is intravitreal injection or implant [20], and the efforts to improve retinal drug delivery are heavily focused on two aspects. The first is slow-release, long-acting ocular drug formulations/implants for macromolecules (such as anti-VEGF (vascular endothelial growth factor)) or steroids that increase the duration of action or reduce the frequency of injection. Recently, a second aspect has emerged that concentrates on more targeted routes of delivery, such as subconjunctival, suprachoroidal, subretinal, and trans-scleral injections. These new delivery techniques, although potentially less invasive, still require repeated injections into the eye. In addition, ocular injections are usually not suitable for the delivery of small molecules as they tend to have short half-lives (usually less than 10 h) of bioavailability in the target tissues [9,14,21]. Other than in steroid treatments, such as dexamethasone intravitreal implants and triamcinolone intravitreal injections [22,23], there are currently no non-steroidal small molecules approved for use to treat retinal diseases. This is not because small molecules cannot treat such diseases, but rather due to the hindered capacity of the currently available delivery methods. As a matter of fact, small molecules, such as VEGF receptor inhibitors, platelet-derived growth factor receptor inhibitors, tyrosine kinase inhibitors, and complement inhibitors, have been presented in scientific publications and listed in ongoing pipelines as potential treatments of dry/wet age-related macular degeneration (AMD) and other retinal diseases [24,25,26,27]. The route of delivery is often through slow-release invasive implants or oral administration with systemic exposure. 

Here, we describe the discovery of a novel non-invasive ocular delivery platform (NIODP) that successfully delivered drug candidates with high bioavailability and high efficiency not only to the front, but also to the back of the nonhuman primate (NHP) eye through topical administration to the periorbital skin. 

## 2. Materials and Methods

### 2.1. Compounds

**Figure 1:**
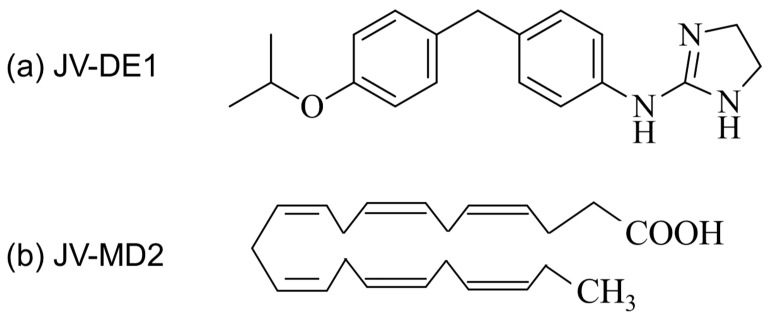
Chemical structures of (**a**) JV-DE1 (RO1138452, CAY10441), 4,5-dihydro-N-[4-[[4-(1-methylethoxy)phenyl]methyl]phenyl]-1H-imadazol-2-amine, formula weight 309.4; and (**b**) JV-MD2 (DHA), cis-4,7,10,13,16,19-docosahexaenoic acid, formula weight 328.49.

The chemical structure of JV-DE1 and JV-MD2 (two small molecules currently under development in the JeniVision pipeline) are presented in Figure 1. JV-DE1 (also known as JV-DE1, RO1138452, CAY10441) was custom synthesized at Raybow Pharmaceutical Science and Technology Co., Ltd., Hangzhou, China. JV-MD2 (DHA free acid) and compound X (described in Section 2.2 and Section 2.6) were purchased from Sigma-Aldrich, St. Louis, MO, USA. These compounds also served as internal standards (IS) for the relative analytical assays.

### 2.2. Formulations

JV-DE1 eye drop formulation: JV-DE1 was dissolved in 2.4% polyoxyl 35 castor oil and 0.2% glycerol in pH7.6 Tris-buffered saline to 2.4 mg/mL (0.24% *w*/*v*). 

Formulations for NIODP application: JV-DE1 was dissolved in medium-chain triglyceride oil to 5.3 mg/g (0.53% *w*/*w*). The original JV-MD2 stock of 98% was diluted in linoleic acid oil pre-dissolved with compound X. The final concentration of JV-MD2 was 215.21 mg/g (21.5% *w*/*w*) and compound X was 16.14 mg/g. A pen brush with a 3-mL reservoir was used as the applicator for each formulation. 

### 2.3. Non-Invasive Ocular Delivery Platform (NIODP)

**Figure 2:**
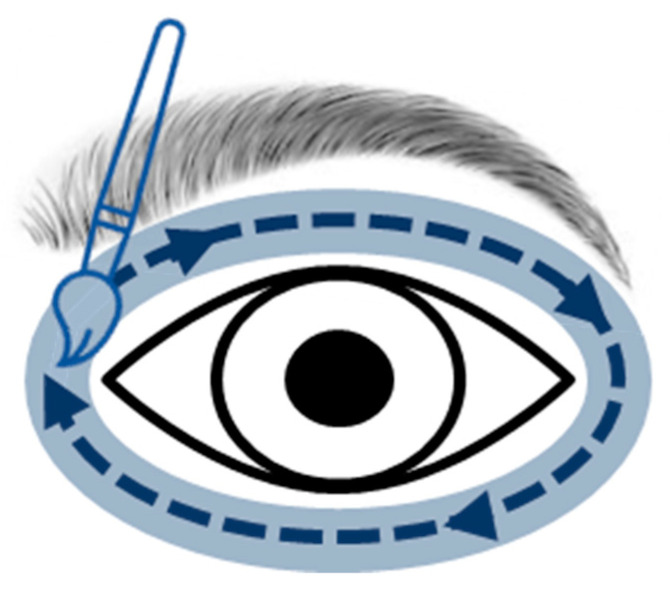
Non-invasive ocular delivery platform (NIODP). A novel drug delivery method to the front and back of the eye via periorbital skin application.

NIODP is a JeniVision proprietary topical ocular drug delivery route via the periorbital skin, i.e., the skin area around the eye orbit, which was first used for glaucoma drug delivery to the anterior chamber of the eye [28]; here, it was discovered to be a novel route for ocular drug delivery, particularly for retinal drugs, as depicted in Figure 2.

### 2.4. Choice of Animals for Eye Drop and NIODP Ocular Biodisposition Studies

A pilot ocular biodisposition study is often utilized in the research and discovery phase, prior to Good Laboratory Practices (GLP) pharmacokinetic studies, to assess the feasibility of the delivery route and reliability of the animal model, as well as to minimize the unnecessary use of animals to be sacrificed, especially in studies of higher species such as non-human primates (NHPs). The biodisposition describes a drug’s distribution (“where”) at a certain time point (“when”). As long as an ocular biodisposition study is designed so that it can answer the questions of “when” and “where” in a generally consistent trend, ideally at multiple time points, such a study design has been accepted by the FDA and for peer-reviewed publications, especially when higher species are used in the studies [6,28,29,30,31].

Rabbits are an ideal model to study topical eye drop formulation delivery, especially to the anterior chamber of the eye. Compared to rodents, rabbits share more common anatomical and biochemical features with humans, such as a larger eye size [32]. Monkeys were chosen for the initial NIODP concept validation as the most suitable animal model due to their hairless periorbital skin, the size of their eyeballs, and the fact that they have a similar ocular anatomy to humans, in order to maximize the usually low animal-to-human translational success rate in non-invasive retinal delivery. Non-naïve NHPs washed out from previously unrelated studies were used for the experiment. For analytical method development and validation, ocular blank matrixes were harvested from animals of a control group after unrelated terminal studies. 

### 2.5. Biodisposition of JV-DE1 Eye Drops in New Zealand Rabbits

Five male New Zealand rabbits aged 4–7 months were used in this study. The animals were housed individually in polypropylene cages (530 mm × 630 mm × 320 mm) and in an environmentally monitored, well-ventilated conventional room maintained at a temperature of 18–26 °C and a relative humidity of 40–70%. A certified rabbit diet was provided ad libitum daily during the quarantine and study periods. The nutritional ingredients and designated chemicals of the diet were analyzed by a qualified institute once quarterly. 

The in-life procedures and sample collections were performed at Joinn Laboratory Inc. (Suzhou, China). A single dose of 50 µL (120 µg) per eye of the 0.24% JV-DE1 eye drops was applied to both eyes of the animals at time 0 using a pipet. This dose was chosen based on the maximum amount deliverable as an eye drop. Blood samples were collected from all five study animals at 0 h pre-dosing. For every time point of 0.5, 2, 4, 8, and 24 h post-dose, one animal was euthanized and the ocular tissues (anterior sclera, aqueous humor, bulbar conjunctiva, ciliary body, cornea, iris, posterior sclera, and retina) of both eyes were collected. Plasma samples were collected before each euthanization.

Sample preparation and bioanalysis were performed at Medicilon Preclinical Research LLC (Shanghai, China). Solid ocular tissues were homogenized with 9- to 49-fold (1 g tissue in 9 mL to 49 mL, *w*/*v*) of cold homogenization buffer (50% methanol/H_2_O). Aliquots of plasma or aqueous humor, as well as the homogenized tissue samples, were mixed with 8-fold volumes of 200 ng/mL warfarin in a methanol solution. After vortexing and centrifuging, an aliquot of supernatants was transferred to a 96-well plate for LC-MS/MS analysis. JV-DE1 is a synthesized new chemical entity (NCE). The absence of the endogenous compound was verified by JeniVision with a battery of pre-clinical GLP and non-GLP studies, including analytical studies in monkey, dog, and rabbit tissues.

### 2.6. Biodisposition of JV-DE1 and JV-MD2 Delivered via NIODP in Non-Human Primates 

Four female cynomolgus non-naïve monkeys aged 3–4 years used for unrelated projects were washed out prior to this study. The monkey maintenance pelleted feed was purchased from Beijing HFK Bioscience Co. Ltd. (Beijing, China) or other qualified sources and was provided ad libitum throughout the in-life portion of the study. During the acclimation period and the experiment, the animals were housed individually (1 monkey/cage) in stainless-steel wire-mesh-type cages in a group housing. 

All experiments were performed at Medicilon. A single dose of about 31.2 mg of the 5.3 µg/mg JV-DE1 formulation, i.e., 165.3 ± 4.6 µg (mean ± SEM) per eye of JV-DE1, was applied to the right eye (OD); and a single dose of about 31.48 ± 0.43 mg of the 215.2 µg/mg JV-MD2 formulation, i.e., 6775 ± 92 µg (mean ± SEM) per eye, was applied to the left eye (OS) of each animal at time 0 via NIODP (this formulation also contained 16.14 µg/mg of compound X (mean 508 µg per OS)), which did not diffuse beyond the periorbital skin. A pen brush was used to dose each formulation with three to four circular motions for even distribution. The doses of the test articles were selected based on their solubility/concentration with the maximum deliverable volume on the periorbital skin of the animals. The administered dose was calculated by the weight difference of the pen brush before and after administration. Animals were restrained from eye rubbing in restraint chairs for 2 h after dosing, with access to food and drink. A blood sample was collected from one of the study animals at pre-dosing. For every time point of 0.5, 3, 6, and 24 h post-dose, one animal was euthanized and the ocular tissues (upper eyelid, cornea, retina, and vitreous humor) of both eyes were collected. Plasma samples were collected before each euthanization. 

For the convenience of tissue processing and sample extraction, only upper eyelids were collected to represent the entirety of the periorbital tissue. Ocular tissues were homogenized by adding a 9-fold volume of 50% methanol/H_2_O per gram of tissue. For the LC-MS/MS sample preparation, a 50 μL aliquot of plasma, aqueous humor, vitreous humor, or tissue homogenate was thoroughly mixed with a 250 μL extraction solution of CHCl3/EtOH (1:1); then, a 210 μL supernatant of the samples was transferred to a 96-well plate after centrifugation. The samples were evaporated in nitrogen gas (N2) until dried. Each sample was redissolved in 200 μL MeOH, of which 2 µL was injected for the LC-MS/MS analysis.

### 2.7. LC-MS/MS Methods for JV-DE1 in Rabbit Plasma and Ocular Tissues

The liquid chromatography (LC) system comprised a Waters (Waters Corporation, Milford, CT, USA) Ultra Performance Liquid Chromatography (UPLC) equipped with an ACQUITY UPLC binary solvent manager, an ACQUITY UPLC sample manager, an ACQUTIY UPLC sample organizer, and an ACQUITY UPLC column heater HT. The mass spectrometric (MS) analysis was performed using a Triple Quad 6500+ (Applied Biosystems/MDS Sciex, Concord, ON, Canada) with an ESI ion source. The data acquisition and control system were created using Analyst 1.6.3 and 1.7.1 Software from Applied Biosystems/MDS Sciex. The LC/MS conditions for JV-DE1 are described in Supplementary Table S1a–c.

### 2.8. LC-MS/MS Methods for JV-DE1 and JV-MD2 in Monkey Plasma and Ocular Tissues

The LC system was the same as that described in Section 2. The MS analysis was performed using a Triple Quad 5500 with an ESI ion source, and the data acquisition and control system were Analyst 1.6.3 software from Applied Biosystems/MDS Sciex. 

The LC/MS conditions for JV-DE1 are described in Supplementary Table S2. The LC conditions were as follows: ACQUITY UPLC ^®^ BEH C18 1.7 μm, 2.1 mm × 50 mm; the mobile phase Column A, 0.1% formic acid in water; and Column B, 0.1% formic acid in acetonitrile. The flow rate was 0.6 ml/min, the column temperature was 50 °C, and the injection volume was 2 µL. The MS conditions were as follows: scan type, positive MRM; ion source, turbo spray; ionization model, ESI; nebulizer gas 1, 55 psi; gas 2, 55 psi; curtain gas, 40 psi; CAD, 10; ionspray voltage, 5500 V; and temperature, 550 °C.

The LC/MS conditions for JV-MD2 (DHA) are described in Supplementary Table S3. The LC conditions were as follows: ACQUITY UPLC^®^ BEH C18 1.7 μm, 2.1 mm × 50 mm; the mobile phase Column A, 10 mm NH_4_AC in H_2_O; and Column B, CAN. The flow rate was 0.5 mL/min, the column temperature was 40 °C, and the injection volume was 4 µL. The MS conditions were as follows: scan type, negative MRM; ion source, turbo spray; ionization model, ESI; nebulizer gas 1, 55 psi; gas 2, 55 psi; curtain gas, 40 psi; CAD, 9; ionspray voltage, 5500 V; and temperature, 550 °C.

## 3. Results

### 3.1. JV-DE1 Ocular and Plasma Bioavailability Delivered by Eye Drops in New Zealand Rabbits: The Evolution of Eye Drop Administration to Glaucoma Drug Periorbital Skin Delivery, to the Discovery of the Non-Invasive Ocular Delivery Platform (NIODP)

The topical periorbital skin route (i.e., via the skin area around the eye orbit) for ocular drug delivery was first used to deliver JV-GL1, a prostanoid EP_2_ receptor agonist, to treat glaucoma, with successful intraocular-pressure-lowering effects in normotensive cynomolgus monkeys [28] and in open-angle glaucoma or ocular hypertension patients in a Phase 1b/2a clinical trial (NCT04761705). Prior to the discovery of the novel topical periorbital skin route, JV-GL1 demonstrated highly effective and long-duration anti-glaucoma effects as eye drops in ocular normotensive monkeys [28].

**Figure 3:**
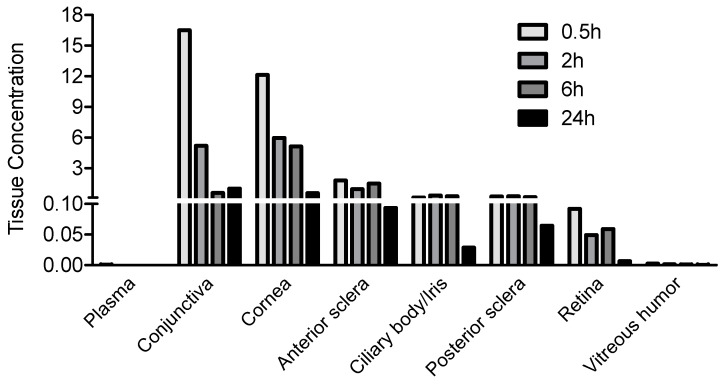
JV-DE1 bioavailability in New Zealand rabbit plasma and ocular tissues when administered as eye drops. A single dose of JV-DE1 (120 µg/eye) was delivered to the right eyes (OD). *N* = four animals in study. The JV-DE1 level is presented as µg/g of solid tissues or µg/mL of aqueous tissues.

JV-DE1 (RO1138452) is an anti-inflammatory dual antagonist of the prostanoid IP receptor and PAF receptor, with a pKi of 9.3 (ki = 0.5 nM) for human IP receptor and 7.9 (Ki = 1.3 nM) for human PAF receptor [33]. It is in development at JeniVision for the treatment of dry eye disease. When a 50 µL JV-DE1 eye drop formulation was administered by pipetting to the right eye (OD) at 120 µg/eye to New Zealand rabbits (Table 1), it accumulated at high concentrations in the ocular surface tissues of the bulbar conjunctiva (Cmax ~16 µg/g) and cornea (Cmax ~12 µg/g) as expected, followed by the anterior sclera (Cmax 1.7856 µg/g), ciliary body/iris (Cmax 0.3049 µg/g), and posterior sclera (Cmax 0.2395 µg/g). The JV-DE1 also reached the retina at a Cmax of 0.0920 µg/g, which is much higher than the 0.0026 µg/g Cmax in vitreous humor. The JV-DE1 eye drop concentration gradient in ocular tissues (Figure 3) was consistent with its maximum ocular delivery efficiency (presented as % administered dose), which was conjunctiva (1.3929%) > cornea (0.8622%) > anterior sclera (0.2982%) > posterior sclera (0.0197%) > ciliary body/iris (0.0174%) > retina (0.0047%) > vitreous humor (0.0023%). There was no significant systemic exposure of JV-DE1 when applied via eye drops, as the drug concentration was no more than 0.001 µg/mL in the plasma throughout the 0.5, 3, and 6 h post-dose time points, with a 24 h clearance.

Although the 0.0047% retinal delivery efficiency of JV-DE1 eye drops in rabbits was about five times higher compared to the maximum of 0.001% efficiency usually seen in most eye drops dosed to lower animal species, a 10- to 100-fold increase in retinal delivery efficiency demonstrated in a large animal species is desirable to ensure clinical success. Since a substantial increase in the drug load is hard to achieve as an eye drop formulation due to limitations related to drug solubility, ocular tolerability, and suitability, the follow-up studies (as described in Section 3.2 and Section 3.3) on ocular drug delivery via the periorbital skin route were undertaken in the hope of achieving pharmaceutically effective retinal doses in larger animal species. 

### 3.2. JV-DE1 Ocular and Plasma Bioavailability after a Single-Dose NIODP Application in Non-Human Primates

**Figure 4:**
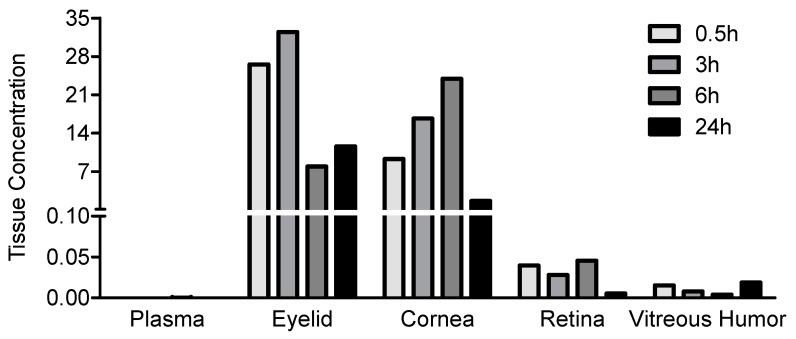
JV-DE1 bioavailability in monkey plasma and ocular tissues delivered via NIODP. A single dose of JV-DE1 was administered to the right eye (OD) at 165.4 µg/eye (mean). Eyelid/periorbital skin is collectively labeled as “Eyelid”. *N* = four animals in the study. The JV-DE1 level is presented as µg/g of solid tissue or µg/mL of fluid samples.

JV-DE1 was topically applied on the periorbital skin at about 165.3 µg/eye to the right eye (OD) of four cynomolgus monkeys (Table 2). A substantial quantity of the drug was found in the eyelid/periorbital skin (Cmax 26.54 µg/g) and cornea (Cmax 9.30 µg/g). On the other hand, the retina (Cmax 0.0455 µg/g) and vitreous humor (Cmax 0.0190 µg/g) exhibited the lowest drug levels. Therefore, the concentration gradient remained similar to the eye drop application, where the dosing site of NIODP (eyelid/periorbital skin) > cornea > retina > vitreous humor (Figure 4). The plasma concentrations remained ≤ 0.0003 µg/mL, near the lower limit of quantitation (LLOQ, 0.0001 µg/mL), showing no significant systemic exposure of JV-DE1 in the current study via NIODP application, similar to the eye drop administration. 

### 3.3. JV-MD2 Ocular and Plasma Bioavailability via NIODP in Non-Human Primates 

JV-MD2 (DHA omega 3 free acid) was administered via NIODP at approximately 6775 µg/eye to the left eye (OS) of four cynomolgus monkeys. The endogenous levels of DHA in the ocular tissues were determined during method validation from the mean of a double-blank matrix measured during method validation, where BLQ is below the limit of quantification, with a lower limit of quantitation (LLOQ) = 0.5 µg/mL. Substantial quantities of JV-MD2 remained in the eyelid/periorbital skin (11–420 µg/g vs. the BLQ baseline), cornea (22–66 µg/g vs. the BLQ baseline), and retina (47–111 µg/g vs. the 17 µg/g baseline), at all post-dose time points of 0.5, 3, 6, and 24 h (Table 3). It is notable that JV-MD2 rapidly reached its Cmax of 111 µg/g in the retina within 0.5 h post-dosage from the endogenous level of 17 µg/g. In the vitreous humor, JV-MD2 remained below BLQ at all timepoints except for at 24 h post-dose (2 µg/mL). The JV-MD2 concentration gradient was as follows: the dosing site of NIODP (eyelid/periorbital skin) > retina > cornea >> vitreous humor. This is generally concordant with the maximum percentage of the administered dose achieved within 30 min of dosing, which was eyelid/periorbital skin (0.4288%) > retina (0.0343%) > cornea (0.0283%) >> vitreous humor. The post-dose plasma levels of JV-MD2 showed a range of 1–3 µg/mL, with no significant fluctuation around the 2 µg/mL pre-dosing baseline. 

## 4. Discussion

According to the tissue concentration gradient (Figure 3) and the % of the administered dose in ocular tissues (Table 1) derived from the rabbit eye drop biodisposition study, most of the JV-DE1 followed a typical “corneal route” of cornea → anterior tissues (ciliary body/iris) → vitreous humor where JV-DE1 lost the driving force to reach the retina; in the meantime, a small portion of the JV-DE1 may also have taken the “conjunctiva–sclera route”, i.e., conjunctiva → sclera → retina. Changing the site of delivery from the ocular surface to the application of eye drops to the periorbital skin around the eye via NIODP did not improve retinal delivery of JV-DE1. Rather, the maximum delivery efficiency in the retina was about 10-fold lower, dropping from 0.0047% via eye drop application to 0.0005% via NIODP, albeit not in a head-to-head comparison, as the studies were conducted in different species. Nonetheless, the tissue concentration gradient again indicated that the JV-DE1 levels achieved in the retina were mainly through the same conjunctiva–sclera pathway as the eye drops. JV-DE1 seems to bypass the vitreous humor to reach the retina, since, in both delivery routes, the retinal bioavailability seems to be higher than that of the vitreous humor. The retinal Cmax of JV-DE1 (RO1138452) was 0.0920 µg/g (297 nM) for eye drop administration in rabbits and 0.0455 µg/g (147 nM) for NIODP in monkeys. This Cmax value is at least 100-fold higher than the Ki on the human receptors of prostanoid IP (ki = 0.5 nM) and PAF (Ki = 1.3 nM) and may be sufficient to treat retinal diseases if the same delivery efficiency is translatable from rabbits to humans as eye drops or from monkeys to humans via NIODP. However, while it may be tolerable to apply the 0.53% (*w*/*w*) oily formulation once daily via NIODP, the 0.24% (*w*/*v*) aqueous eye drop formulation may cause eye irritation in humans.

**Figure 5:**
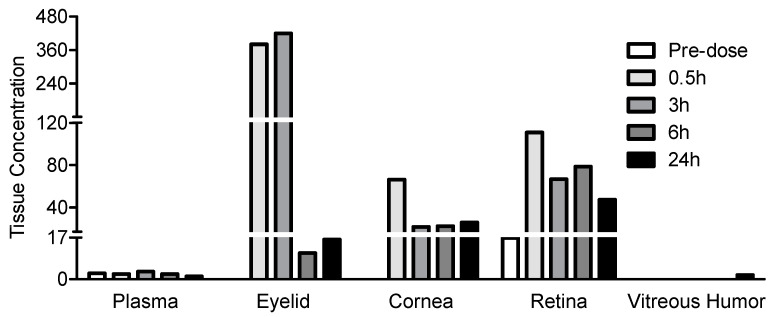
JV-MD2 bioavailability in monkey plasma and ocular tissues delivered via NIODP. A single dose of JV-MD2 was administered at 6775 µg/eye (mean) to the left eye (OS). Eyelid/periorbital skin is collectively labeled as “eyelid”. *N* = four animals in the study. The JV-MD2 level is presented as µg/g of solid tissue or µg/mL of fluid sample.

Although both molecules are amphipathic, JV-DE1 can form an aqueous formulation, while JV-MD2 (DHA) is a fatty acid with much lower water solubility. This difference in physicochemical properties makes it easier for DHA to penetrate cell membranes and diffuse through biological tissues to achieve efficient transcellular and intracellular distributions. We have discovered a novel non-invasive ocular delivery platform, and demonstrated that the application of less than 7 mg of JV-MD2 (DHA) via NIODP can reach a Cmax of 66µg/g in the cornea and 111 µg/g in the retina, where the delivery efficiencies were 0.01–0.42% in the eyelid/periorbital skin (site of application), 0.01–0.03% in the cornea, and 0.01–0.04% in the retina of NHPs. Like JV-DE1 (administered either as eye drops or via NIODP), JV-MD2 seems to reach the retina via the conjunctiva–sclera pathway when administered via NIODP, with a preferred distribution in the retina over the cornea (Figure 5). Interestingly, the biodistribution of JV-MD2 only appeared in the vitreous humor at 24 h post-dose but not at any earlier time points of 0.5, 3, or 6 h post-dose; moreover, there was no endogenous DHA detected in the blank matrix. Thus, excess JV-MD2 may diffuse from the retina to vitreous humor over time if other reasons, such as animal-to-animal variation, can be ruled out.

In contrast to traditional eye drop administration, which targets the cornea mostly for anterior ocular drug delivery, the conjunctival–scleral pathway has not previously been considered a major drug delivery route, although ocular drug penetration may occur via this pathway. The conjunctival–scleral pathway may provide a much larger surface area for drug absorption than the cornea [34]. Compared to the cornea, the relatively leaky and hydrophilic conjunctival tissue can provide approximately 230-fold larger intercellular spaces that are more permeable even to macromolecules, such as proteins and peptides [3]. Connected to the conjunctiva is the sclera, a network of collagen fibers, proteoglycans, and glycoproteins in an aqueous medium forming scleral water channels 30–350 nm in size [9,12], spacious enough for the passage of macromolecules. The suprachoroid is located between the sclera and the choroid. Since the choroid bleeds into the suprachoroid space (SCS), the exchange of biomolecules between the choroid, SCS, and sclera is barrier-free. Because of this permeability, as well as being less invasive and having higher bioavailability than intravitreal injection, suprachoroidal injection has been under investigation since 2013 for retinal delivery [35,36,37]. The fenestrated capillaries in the choroid are highly permeable and allow for high concentrations and the rapid diffusion of nutrients in the extra-vascular space of the choroid [38]. Bruch’s membrane (BrM) is a thin layer of extracellular matrix, which is a selectively permeable membrane between the retina and choroid and regulates the exchange of nutrients, oxygen, minerals, and by-products of the visual cycle through passive diffusion, influenced by the weight, size, and shape of the diffusing molecule. While some complement proteins, such as FHL-1, factor D, and C5a, are allowed to diffuse through, most complement proteins (including the low-molecular-weight C3a) are unable to do so [39,40]. Finally, before any drug reaches the retina, it must pass the retinal pigment epithelium (RPE), which also forms the outer blood–retina barrier (BRB), regulating drug permeability via physicochemical properties, such as molecular weight, lipophilicity, protein binding, and concentration gradient [12].

**Figure 6:**
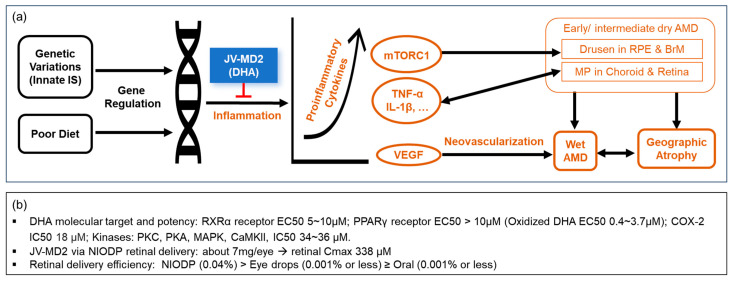
MOA and pharmacological effects of DHA on AMD. (**a**) The anti-inflammatory effects of DHA on drusen genesis, proinflammatory cytokine secretion, and macrophage accumulation in the choroid and retina. (**b**) DHA molecular target, potency, and retinal delivery efficiency via various routes. IS, immune system; RPE, retinal pigment epithelium; BrM, Bruch’s membrane; MP, macrophages.

Age-related macular degeneration (AMD) in the elderly is the leading cause of irreversible vision loss. Early or intermediate stages of AMD (dry AMD) are defined by the formation of lipid-rich deposits of drusen between the RPE and the BrM, as well as the accumulation of choroidal macrophages. Geographic atrophy (GA), one of the advanced stages of AMD, is characterized by the confluent deterioration of the RPE and photoreceptor and choroidal neovascularization. Wet (or neovascular) AMD, another advanced stage of AMD, is characterized by invasive choroidal neovascularization (CNV) with accompanying macrophage accumulation in the retina, which breaks from the BrM and can lead to retinopathy [41]. Genetic variations in the innate immune system and poor diet are the two main factors contributing to drusen genesis and disease progression in AMD [42]. The mechanistic target of rapamycin (mTOR), a sensor of nutrient availability and growth factors, has been implicated in multiple diseases like cancer, diabetes, and neurodegenerative diseases; they are characterized by inflammation, as well as aging, referred to as “inflammaging” [43,44,45]. The activation of mTORC1 has been associated with the formation of drusen-like deposits in photoreceptors; dietary supplementation with DHA alleviated most pathologies in a mouse model with advanced AMD pathologies [42]. The once-daily intragastric administration of DHA effectively inhibited laser-induced CNV formation in mice, which was associated with the suppressed protein expression of NF-κB, VGFER2, and VEGF, major players in the angiogenic pathway in cancer and advanced stages of AMD [46]. Upon the induction of pathological stimuli, activated macrophages were recruited to the affected site, i.e., the back of the eye in the case of AMD, to secrete proinflammatory cytokines, recruit more macrophages, and accelerate disease progression. Omega 3 fatty acids provoke major alterations in gene expression in macrophages to decrease cytokine production while increasing phagocytosis to eliminate pathogens. It is worth noting that the anti-inflammatory effect mediated by DHA was more potent than that of EPA; the only cytokine secretion increased by the omega 3 fatty acid treatment was the anti-inflammatory cytokine IL-10, while many other cytokines, such as IL-1β, TNF-α, and IL-6, were found to be decreased in omega-3-fatty-acid-treated macrophages [47]. Therefore, the mechanism of action of DHA in early and intermediate dry AMD treatments is thought to involve the suppression of drusen genesis via mTORC inhibition, the alteration of the function of macrophages toward an anti-inflammation phenotype by inhibiting proinflammatory cytokine production, and the mediation of anti-neovascularization effects by inhibiting the effects of VEGF in the choroid and retina. Given the success in treating early- or intermediate-stage dry AMD by DHA, it is logical to anticipate the prevention of late-stage AMD, wet AMD, and geographic atrophy, which have prevalence levels of approximately 10% of all individuals with AMD [48,49] (Figure 6a).

Regarding human clinical trials for dry AMD treatment, in 2015, Georgiou and Prokopiou reported preliminary but promising therapeutic results of taking 5 g/day omega 3 in patients with mild-to-moderate visual impairment of dry AMD [50]. Then, in 2022, in an ARVO annual meeting abstract, the same group reported that 3.7 g daily oral administration of omega 3 fatty acids improved objective and subjective vision in patients with dry AMD and SD [51]. A peer-reviewed article has not yet been published. In a three-year randomized clinical study of patients with early lesions of AMD, the omega-3-supplemented (840 mg/day DHA and 270 mg/day EPA) patients who had high red blood cell membrane EPA and DHA levels were significantly protected against choroidal neovascularization of AMD compared with those in the olive oil placebo group [52]. Additionally, the preventative effect of omega 3 in AMD has clearly been demonstrated in a large number of epidemiological studies, using different methodologies, populations, and geographical sites with a high degree of consistency [53]. However, no interventive treatment effect from omega 3 consumption was found in patients with advanced AMD [54]. This may be due to two reasons: (a) the poor absorption and poor retinal delivery efficiency of orally administered omega 3, even with mega dosage [7,53,55]; and (b) the pharmaceutically effective doses for disease treatment are usually expected to be at least a few times higher than the doses for disease prevention, where normal physiological levels are good enough for maintaining health. For example, the European Food Safety Authority (EFSA) suggested that the amount of EPA+DHA required to lower triglyceride is 2–4 g/day and 3 g/day to lower blood pressure, which are, respectively, about 4- to 13-fold higher than the daily consumption recommended to stay healthy according to the World Health Organization (WHO) [56]. 

A healthy retina contains a high concentration of DHA (the active ingredient of JV-MD2), which is not only important in the maintenance of normal retinal integrity and visual function, but also plays anti-inflammatory, anti-apoptotic, and neuroprotective roles [56,57]. DHA analogs may act as anti-inflammatory lipid mediators to activate peroxisome-proliferator-activated receptors (PPARs) and retinoid X receptors (RXRs). As a natural ligand, DHA induced a protective effect in rat retinal neuronal cultures to promote the survival and differentiation of photoreceptors by activating RXRs and the downstream signaling pathways [58]. The EC_50_ of DHA was about 5–10 μm for human RXRα receptor, and an oxidized form of DHA can be as potent as an EC_50_ of 0.4 μM for human PPARγ [59,60]. It was suggested that DHA and its more potent metabolites compete with the more biologically potent arachidonic acid on the COX-2 (IC_50_ 18 μM) signaling cascade and can shift the proinflammatory state to a more anti-inflammatory one [61,62]. DHA was able to suppress inflammation through reducing IL-1β production with a potency of IC_50_ = 4.6 µM in the human THP-1 macrophage cell line [63]. DHA was also found to reduce the activity of protein kinase C (PKC), cAMP-dependent protein kinase A (PKA), mitogen-activated protein kinase (MAPK), and Ca21/calmodulin-dependent protein kinase II (CaMKII) at an IC_50_ of 34–36 µM in in vitro functional assays [64]. For retinal disease treatments, a local drug concentration 3- to 10-fold higher than EC_50_ or IC_50_ is desirable, which may be readily achieved in the retina utilizing the NIODP, as demonstrated in our current study, where the application of just 7 mg/eye of DHA in NHP yielded a retina DHA Cmax of 111 µg/g (about 338 µM) (Figure 6b). This JV-MD2 retinal Cmax is at least 10-fold higher than the EC_50_ or IC_50_ of most mediators in the omega 3 DHA signaling cascade. Such concentration is believed to be enough to treat retinal diseases because the success of drug treatments lies in the effective drug concentration deliverable to the target tissue [65]. Moreover, we believe that much higher DHA retinal concentrations can be achieved via NIODP using formulations with a higher concentration and more frequent dosing, if necessary. 

Because of the “500 Dalton rule” for passive skin penetration [66], small molecules are the best candidates for NIODP delivery. However, liposomes up to 600 nm in diameter have been reported to penetrate the skin through intercellular lipids of the stratum corneum [67,68]. Liposomes with an average size of 300 nm in diameter were reported to penetrate deeper into skin layers, and those with a 70 nm diameter showed the best dermal delivery performance [69,70]. For reference, the average molecular widths are 2.5 nm for DNA, 10 nm for proteins, 100 nm for a typical virus, and 1000 nm for a bacterium [71]. A previous publication indicated that liposomes can deliver macromolecules through the skin [72]. In posterior ocular delivery, nanocarriers have been reported to overcome the ocular barriers, as nanoparticles <250 nm were usually easily taken up by retinal cells via endocytosis [9,73]. Therefore, it is possible that NIODP may have the potential to be utilized for macromolecular therapeutics packed in liposomes for retinal delivery. More vigorous research is necessary.

**Figure 7:**
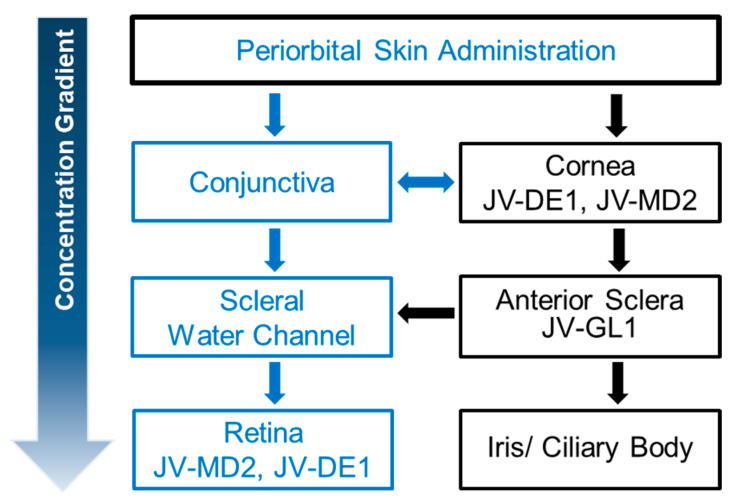
Proposed concentration gradient of drug distribution administered through NIODP and drug candidates found in the targeted tissue. The JV-GL1 biodistribution is unpublished (JeniVision data).

The NIODP via periorbital skin application is also a novel route for anterior ocular drug delivery. It has successfully delivered JV-GL1, a prostaglandin EP2 receptor agonist, for glaucoma treatment in a phase 1b/2a clinical trial with satisfactory efficacy, while avoiding ocular adverse effects commonly associated with prostaglandin eye drops directly applied to the ocular surface. It is important to note that, for issues of solubility or ocular tolerability, most hydrophobic vitamins and supplements, including omega 3, cannot be conveniently used as high-dose eye drops. The NIODP makes it possible to deliver these molecules for the potential treatment of anterior ocular diseases, such as dry eye disease, uveitis, etc., with minimum irritation or interference with normal ocular functions. A summary of the therapeutic agents (JV-GL1, JV-DE1, and JV-MD2) that were successfully delivered through periorbital skin administration to the targeted tissues is shown in Figure 7.

Notably, like most eye drops used in a range of reasonable ocular doses (0.01–0.5%), the systemic exposure of JV-DE1 was very low when using both eye drops and NIODP as delivery routes with a 24 h clearance. Additionally, when delivered as a single dose at about 7 mg per eye via NIODP, JV-MD2 also did not significantly change the baseline omega 3 levels in the plasma. Therefore, while achieving breakthrough retinal delivery efficiency, the systemic exposure via NIODP application may not pose a great concern; this may also be determined by the physicochemical properties and biosafety profile of the drug.

Similar to JV-DE1 applied via eye drops, both JV-DE1 and JV-MD2 applied via NIODP seem to reach the retina via the conjunctiva–sclera pathway (Figure 7). The potential advantages of NIODP include (a) a breakthrough in non-invasive, high-efficiency retinal delivery; (b) a much more ocularly tolerable delivery method than eye drops for therapeutic agents known to cause ocular adverse effects or interfere with normal functioning of the eye, such as drug ingredients that cause eye irritation due to ocularly intolerable drug property or concentration, or oily formulations causing vision blurriness and eye stress; and (c) another topical ocular delivery route without significant systemic exposure, just like the traditional eye drop administration, but potentially more convenient for self-administration or administration by a care-giver. 

Like any other delivery route (oral, intravenous injection, eye drop, or intravitreal injection), NIODP delivery has its prerequisite conditions of use. However, such limitations do not discount its value as a novel non-invasive ocular delivery platform, which is not just specific to DHA or JV-DE1. A successful NIODP delivery is determined by the physicochemical properties of the drug; it must (a) have permeability through the periorbital skin, which is determined by its molecular weight, lipophilicity, and solubility; (b) have good solubility/concentration to establish a sufficient concentration gradient to drive efficient, high-bioavailability diffusion from the anterior to posterior ocular tissues; and (c) be compatible with the local tissue, such as the sclera water channel, in order to travel through and reach the retina.

In summary, any drug that can penetrate the periorbital skin and can diffuse through ocular tissues may be deliverable by NIODP, which is presented in this report as a novel general delivery platform for ocular delivery to both the anterior and posterior segments of the eye. The site of periorbital skin application enables the utilization of the conjunctiva–sclera pathway for a much higher retinal delivery efficiency using NIODP compared to eye drop delivery. Good bioavailability is driven by the concentration gradient of ocular tissues, where the drug solubility plays an important role when the delivery volume is fixed. Due to the “500 Dalton rule” for passive skin penetration, small molecules are the best candidates for NIODP delivery. With more thorough research, it may be possible to utilize NIODP for the retinal delivery of macromolecular therapeutics packed in liposomes or other nanocarriers. Nonetheless, our studies demonstrated the potential of NIODP for use in the high-efficiency drug delivery of small molecules for the treatment of various ocular disorders, particularly for retinal diseases.

## 5. Conclusions

The discovery of a novel non-invasive ocular delivery platform has opened up new possibilities for the effective topical delivery of molecules that are currently difficult to deliver to both the front and back of the eye. The NIODP is a combination of periorbital skin administration with topical drug formulation for ocular, particularly retinal, delivery. JV-MD2 (DHA) has been successfully delivered by NIODP with a high efficiency and high bioavailability to the retina. When the essential fatty acid DHA, with a multi-anti-inflammatory mechanism of action, reaches the retina at a concentration up to 10-fold higher than the normal EC_50_ or IC_50_ of its molecular targets, a better therapeutic efficacy can be anticipated for retinal diseases (such as early or intermediate AMD, thus preventing late-stage AMD) and avoiding or minimizing the frequency of invasive ocular injections. With further study and more experimental evidence, the NIODP could provide significant opportunities to address the serious medical need in drug delivery for the treatment of eye diseases without irritation or injection, transforming the retinal health landscape. 

## 6. Patents

Patent (pending) resulting from the work reported in this manuscript: Delivery methods for Treating Eye Diseases, by W.W., D.W., and N.S.

## Figures and Tables

**Table 1:** JV-DE1 in New Zealand rabbits following the administration of a single eye drop in the right eye.

Test Article		JV-DE1 (0.24% *w*/*v*)				
Dosing site		Topical eye drop (OD)				
Rabbit ID		All	2036281	2036282	2036283	2036284
Time (h)		0	0.5	3	6	24
Dose level (μg/eye)		0	120	120	120	120
Drug in plasma (μg/mL)		BLQ	0.0010	0.0003	0.0001	BLQ
Drug in vitreous humor (μg/mL)		NF *	0.0026	0.0017	0.0012	0.0004
Drug in OD Tissues (μg/g)	Conjunctiva	NF *	16.4919	5.1781	0.5660	0.9937
	Cornea	NF *	12.1249	5.9526	5.1255	0.5422
	Anterior sclera	NF *	1.7856	0.9263	1.4785	0.0932
	Ciliary body/iris	NF *	0.1054	0.3049	0.2314	0.0289
	Posterior sclera	NF *	0.2090	0.2395	0.1512	0.0645
	Retina	NF *	0.0920	0.0491	0.0589	0.0068
	Vitreous humor (μg/mL)	NF *	0.0026	0.0017	0.0012	0.0004
% Administered Dose	Conjunctiva		1.3929	0.4477	0.1288	0.1467
	Cornea		0.8622	0.4239	0.3709	0.0254
	Anterior sclera		0.2982	0.1068	0.2139	0.0127
	Ciliary body/iris		0.0084	0.0174	0.0137	0.0021
	Posterior sclera		0.0153	0.0323	0.0197	0.0088
	Retina		0.0047	0.0036	0.0037	0.0004
	Vitreous humor		0.0023	0.0012	0.0007	0.0004

OD: right eye; BLQ: below the limit of quantification, given a value of 0 in the relevant calculations; LLOQ: lower limit of quantitation, equal to 0.0001 µg/mL, given a value of 0 in the relevant calculations thereafter. Delivery efficiency = % of administered dose = 100X (drug content in tissue (μg))/dose level (μg/eye). NF *: not found in undosed animals of various preclinical species, as JV-DE1 is a synthetic novel chemical entity.

**Table 2:** JV-DE1 in cynomolgus monkeys following a single-dose administration via NIODP.

Test Article		JV-DE1 (0.53% *w*/*w*)				
Dosing site		Periorbital skin (OD)				
Animal no.		101	101	102	103	104
Time (h)		0	0.5	3	6	24
Dose level (μg/eye)		0	179.1	160.6	162.2	159.3
Drug in plasma (μg/mL)		BLQ	0.0001	0.0002	0.0003	BLQ
Drug in OD Tissues (μg/g)	Eyelid/periorbital skin	NF *	26.5354	32.4547	7.9456	11.5978
	Cornea	NF *	9.2993	16.6887	23.9295	1.6213
	Retina	NF *	0.0396	0.0279	0.0455	0.0055
	Vitreous humor (μg/mL)	NF *	0.0152	0.0079	0.0039	0.0190
% Administered Dose	Eyelid/periorbital skin		0.6370	1.4956	0.3477	0.7501
	Cornea		0.1921	0.3430	0.5900	0.0326
	Retina		0.0004	0.0004	0.0005	0.0002
	Vitreous humor		0.0047	0.0029	0.0031	0.0018

OD: right eye; BLQ: below the limit of quantification, given a value of 0 in the relevant calculations. LLOQ: lower limit of quantitation, equal to 0.0001 µg/mL, given a value of 0 in the relevant calculations thereafter. Delivery efficiency = % of administered dose = 100X (drug content in tissue (μg))/dose level (μg/eye). NF *: not found in undosed animals of various preclinical species, as JV-DE1 is a synthetic novel chemical entity.

**Table 3:** JV-MD2 in cynomolgus monkeys after a single-dose administration via NIODP.

Test Article		JV-MD2 (DHA (21.5% *w*/*w*)				
Dosing site		Periorbital skin (OS)				
Animal no.		101	101	102	103	104
Time (h)		0	0.5	3	6	24
Dose level (μg/eye)		0	6568	6768	7018	6747
Drug in plasma (µg/mL)		2	2	3	2	1
Drug in OS Tissues (µg/g)	Eyelid/periorbital skin	BLQ *	381	420	11	16
	Cornea	BLQ *	66	22	22	26
	Retina	17 *	111	67	79	47
	Vitreous humor	BLQ *	0	0	0	2
% Administered Dose	Eyelid/periorbital skin		0.4288	0.3844	0.0149	0.0197
	Cornea		0.0283	0.0109	0.0149	0.0173
	Retina **		0.0343	0.0264	0.0228	0.0175
	Vitreous humor		0.0000	0.0000	0.0000	0.0236

OS: left eye; BLQ: below the limit of quantification, given a value of 0 in relevant calculations. LLOQ: lower limit of quantitation, equal to 0.5 µg/mL, and given a value of 0 in relevant calculations thereafter. Delivery efficiency = % of administered dose = 100X (drug content in tissue (μg))/dose level (μg/eye). * Mean of calculated endogenous DHA from the double-blank matrix measured during method validation. ** Endogenous DHA was subtracted when calculating % administered dose in the retina.

## Data Availability

Data are available on request due to restrictions related to privacy and confidentiality.

## Supplementary Materials

The following supporting information can be downloaded at: https://www.mdpi.com/article/10.3390/pharmaceutics15092344/s1, Table S1a: LC conditions for JV-DE1 in rabbit plasma samples. Table S1b: LC conditions for JV-DE1 in rabbit ocular tissues. Table S1c: MS conditions for JV-DE1 in rabbit plasma and ocular tissues. Table S2: LC/MS process conditions for JV-DE1 in monkey plasma and ocular tissues. Table S3: LC/MS process conditions for JV-MD2 (DHA) in monkey plasma and ocular tissues.
