# Supplementary material for: Discovery and Potential Utility of a Novel Non-Invasive Ocular Delivery Platform
Source: Pharmaceutics. 2023 Sep 19;15(9):2344. doi: 10.3390/pharmaceutics15092344 (PMC10535219; doi:10.3390/pharmaceutics15092344)
Supplement: Supplementary file 1 [file pharmaceutics-15-02344-s001.zip › pharmaceutics-2569012-supplementary.pdf]

Supplementary Information

Supplementary Table S1a. LC conditions for JV-DE1 in rabbit plasma samples

Items	Detail Information		
Column	Waters BEH C18 1.7μm (50×2.10mm)		
Column temperature	50 °C		
Auto-sampler temperature	4 °C		
Mobile Phase	MPA: 0.1% Formic Acid (FA) in water<br>MPB: 0.1% Formic Acid (FA) in acetonitrile (ACN)		
Gradient elution	Flow rate	0.6ml/min	
	Time/min	MPA (%)	MPB (%)
	Initial	75.0	25.0
	1.30	5.0	95.0
	1.75	5.0	95.0
	1.76	75.0	25.0
	2.00	75.0	25.0
Injection Volume	3μL		

Supplementary Table S1b. LC conditions for JV-DE1 in rabbit ocular tissues

Items	Detail Information		
Column	Waters BEH C18 1.7μm (50×2.10mm)		
Column temperature	50 °C		
Auto-sampler temperature	4 °C		
Mobile Phase	MPA: 0.1% FA and 1mM NH_4Ac in H_2O/ACN (95:5, v:v)<br>MPB: 0.1% FA and 1mM NH_4Ac in H_2O/ACN (5:95, v:v)		
Gradient elution	Flow rate	0.6ml/min	
	Time/min	MPA (%)	MPB (%)
	Initial	60.0	40.0
	1.00	2.0	98.0
	1.30	2.0	98.0
	1.31	60.0	40.0
	1.50	60.0	40.0

Items	Detail Information
Injection Volume	2μL
Valco Valve	0.3min: switch into mass, 0.8min: switch into waste

Supplementary Table S1c. MS conditions for JV-DE1 in rabbit plasma and ocular tissues

Items	Detail Information						
Ion Source	Ion Spray Voltage: 5500V						
	CAD: 10						
	CUR: 40psi						
	Gas1: 55psi						
	Gas2: 55psi						
	Temperature: 450°C (plasma); 550°C (eye tissue and aqueous humor)						
	Resolution Q1: unit						
	Resolution Q3: unit						
	MR Pause: 5.007msec						
Model	ESI, Positive, MRM						
Mass Parameters (plasma)	Analyte and IS	Q1/Q3	Dwell time (ms)	DP (v)	EP (v)	CE (v)	CXP (v)
	JV-DE1	309.8/266.3	150	150	10	46	13
	Tolbutamide	271.1/172.0	150	70	10	18	13

Mass Parameters (eye tissue and aqueous humor)	Analyte and IS	Q1/Q3	Dwell time (ms)	DP (v)	EP (v)	CE (v)	CXP (v)
	JV-DE1	309.8/266.3	80	150	10	46	13
	Tolbutamide	271.1/172.0	80	70	10	18	13

Supplementary Table S2. LC/MS process conditions for JV-DE1 in monkey plasma and ocular tissues

LC conditions:

Time/min	Flow rate (ml/min)	A%	B%
Initial	0.6	75	25
1.30	0.6	5	95
1.75	0.6	5	95
1.76	0.6	75	25
2.00	0.6	75	25

Supplementary Table S3. LC/MS process conditions for JV-MD2 (DHA) in monkey plasma and ocular tissues.

LC conditions:

Time/min	Flow rate (ml/min)	A%	B%
Initial	0.5	40	60
2.20	0.5	10	90
2.50	0.5	10	90
2.51	0.5	40	60
2.90	0.5	40	60
